# Intestinal Colonization Dynamics of *Vibrio cholerae*

**DOI:** 10.1371/journal.ppat.1004787

**Published:** 2015-05-21

**Authors:** Salvador Almagro-Moreno, Kali Pruss, Ronald K. Taylor

**Affiliations:** Department of Microbiology and Immunology, Geisel School of Medicine at Dartmouth, Hanover, New Hampshire, United States of America; Stony Brook University, UNITED STATES

## Abstract

To cause the diarrheal disease cholera, *Vibrio cholerae* must effectively colonize the small intestine. In order to do so, the bacterium needs to successfully travel through the stomach and withstand the presence of agents such as bile and antimicrobial peptides in the intestinal lumen and mucus. The bacterial cells penetrate the viscous mucus layer covering the epithelium and attach and proliferate on its surface. In this review, we discuss recent developments and known aspects of the early stages of *V*. *cholerae* intestinal colonization and highlight areas that remain to be fully understood. We propose mechanisms and postulate a model that covers some of the steps that are required in order for the bacterium to efficiently colonize the human host. A deeper understanding of the colonization dynamics of *V*. *cholerae* and other intestinal pathogens will provide us with a variety of novel targets and strategies to avoid the diseases caused by these organisms.

## Introduction

The gram-negative bacterium *Vibrio cholerae* O1 is the etiological agent of epidemic cholera, a severe diarrheal disease. Cholera has devastated civilizations throughout history, and, to date, seven pandemics have been recorded. The most recent pandemic still affects millions of people and causes more than 100,000 deaths every year. In recent times, the bacterium has become endemic in places that had been cholera-free for centuries [1]. For instance, since the introduction of *V*. *cholerae* in Haiti after the 2010 earthquake, more than 700,000 people have contracted cholera, resulting in more than 8,500 deaths [2,3].

**Fig 1:**
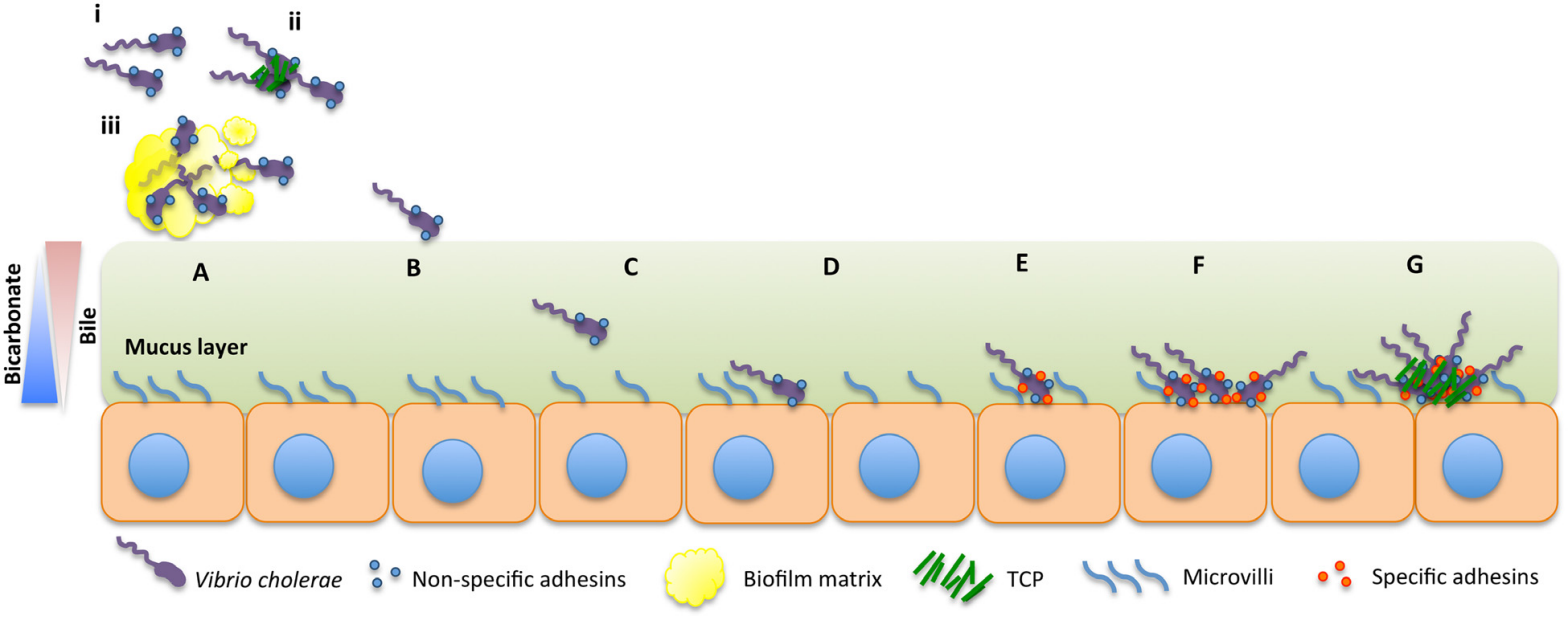
Model for intestinal colonization dynamics of *V*. *cholerae*. *V*. *cholerae* may be ingested as free-living cells (i), as forming microcolonies (ii), or as part of a biofilm (iii) (A). Cells in the lumen will first come in contact with the mucus layer (B). The bacterium must reach the intestinal epithelium by penetrating through the viscous mucus layer covering it (C). Once the bacterium reaches the intestinal epithelium, we hypothesize that noncommitted (reversible) attachment occurs, mediated by adhesins such as GbpA or Mam7 (D). Subsequently, specific attachment adhesins might be produced that would allow *V*. *cholerae* to bind in a committed fashion (E), the cells multiply (F), and, once a certain concentration of cells has been reached, the toxin coregulated pilus is produced, allowing for microcolony formation and toxin production (G).

*V*. *cholerae* is a natural inhabitant of aquatic environments, such as rivers, estuaries, and oceans, where it can be found as free-living cells or attached to biotic or abiotic surfaces [4,5]. Epidemic cholera is transmitted to humans by consumption of water or food contaminated with virulent strains of *V*. *cholerae* O1 [1,6]. Recently, there have been significant advances in the understanding of some key steps in the early stages of colonization of the small intestine (SI) by *V*. *cholerae*. Here, we review these developments and propose a model for the colonization dynamics of *V*. *cholerae* (Fig 1), suggesting mechanisms to fill the gaps in our current knowledge.

## Initial Stages of Colonization

### Relying on and then relinquishing protection

*V*. *cholerae* has a complex acid tolerance response involving numerous factors such as the ToxR-regulated porin, OmpU, the transcriptional regulators CadC and HepA, the gluthatione synthetase GshB, and the DNA repair and recombination enzyme RecO, among others [7–9]. To date, the roles of OmpU and CadC have been corroborated by in-frame deletions [8,10]. Free-living *V*. *cholerae* cells are very sensitive to the low pH of the stomach, and the dose required to cause infection in healthy volunteers, 10^11^ cells, is perhaps unrealistically high [11]. However, when the pH of the stomach is buffered, the number of cells required to cause the symptoms of the disease can be reduced by several orders of magnitude, between 10^4^–10^6^ cells (Fig 1A) [11,12]. Furthermore, in endemic regions, some cholera patients have been found to have low gastric acid production, indicating that these individuals might be more susceptible to free-living *V*. *cholerae* than others [13–15]. With further respect to the physiological state of the bacteria, *V*. *cholerae* might also enter the human host in a dormant state called viable but nonculturable (VBNC) [16–19]. VBNC cells in other species have been shown to have increased acid tolerance [20]. *V*. *cholerae* VBNC cells were given to human volunteers, and these cells were able to effectively colonize the SI and were shed as culturable free-living cells [18].

*V*. *cholerae* might also be ingested as microcolonies or in a hyperinfectious state [21–23]. Once shed after intestinal colonization, *V*. *cholerae* cells can be found in a hyperinfectious state that is thought to lower the infectious dose required to colonize secondary individuals [21]. Furthermore, after infection, subpopulations of *V*. *cholerae* keep expressing the gene encoding TcpA, a major component of the toxin-coregulated pilus (TCP), an essential intestinal colonization factor [22,23]. Microcolonies are TCP-mediated clusters of *V*. *cholerae* cells that confer numerous properties to the bacterium (See section “Final Stages of Colonization”). It is possible that microcolonies shed from cholera patients might confer resistance to the low pH of the stomach to *V*. *cholerae*. However, to our knowledge, the role of microcolonies in low pH tolerance and how the bacterium relinquishes them upon arrival in the SI remain to be determined (Fig 1A).

Biofilms are bacterial communities that collectively produce a protective exopolysaccharide matrix, which facilitates survival during stress-inducing environmental changes such as low pH or the presence of antimicrobials [24]. *V*. *cholerae* that are ingested as part of a biofilm can successfully survive the low pH of the human stomach [25]. Cells within a biofilm may reach the stomach either attached to a substrate or as conditionally viable environmental cells (CVEC)—clumps of dormant cells embedded in a biofilm matrix that can be recovered using enriched culturing techniques (Fig 1A) [25]. Furthermore, while forming biofilm, *V*. *cholerae* can be found in a hyperinfectious physiological state [26]. The infectious dose for biofilm-derived *V*. *cholerae* is orders of magnitude lower than that of planktonic cells regardless of whether the biofilm is intact or dispersed [26]. The relationship between bile and biofilm remains contested [27,28]. Hung and Mekalanos showed that bile stimulates biofilm formation in *V*. *cholerae* as biofilms increase the resistance of the bacterium to bile acids [27]. Conversely, it was recently found that taurocholate, a component of bile, induces the degradation of *V*. *cholerae* biofilms [28]. The authors suggested that contact with bile components upon reaching the intestinal lumen might allow for the dispersal of the bacterium in the early stages of colonization (Fig 1A) [28]. Once in the lumen, the bacterium must withstand the presence of antimicrobial agents. It has been shown that OmpU protects against bile acids [29] and antimicrobial peptides [30] among others.

Overall, it is possible that in the early stages of cholera epidemics, *V*. *cholerae* might be primarily ingested attached to surfaces while forming biofilms, such as the chitinaceous shell of copepods, as CVEC or as VBNC [4,5,31–34]. However, once the cholera epidemic begins, the bacterium might be predominantly consumed as part of microcolonies shed by other cholera patients or in a hyperinfectious state [21].

## Contact with and Swimming through the Mucus Layer

### Directionality towards the epithelium

Motility has been shown to be a crucial element in order for *V*. *cholerae* to colonize the epithelium and cause a successful infection of the human host (Fig 1C) [35,36]. Early studies by Guentzel et al. suggested that motility could enable *V*. *cholerae* to penetrate the mucus layer covering the intestinal epithelium, as nonmotile mutants showed reduced virulence [37]. Nonetheless, it was recently shown that, even though motility is critical for colonization of the proximal SI, motility is not required for the colonization of the distal section of the SI [38]. It is possible that motility enables the dissemination of *V*. *cholerae* throughout the lumen of the SI and other nonflagellum-based processes might control its penetration into the intervillous space [38,39].

The possible role of chemotaxis in establishing a productive infection remains debated. Motile, but nonchemotactic, mutants of *V*. *cholerae* outcompete wild-type *V*. *cholerae* in the infant mouse model [36,38,40]; 10-fold fewer nonchemotactic *V*. *cholerae* are required for infection than wild type [41]. It appears that the competitive advantage of the nonchemotactic mutants is the result of an alteration in the bias of flagellar rotation from clockwise to counterclockwise [41]. Whereas wild-type *V*. *cholerae* predominantly colonizes the distal half of the SI, nonchemotactic mutants are distributed throughout the SI [36]. A recent study by Millet et al. demonstrated that the specific localization in the SI of nonchemotactic mutants does not differ from that of wild type [38]. Thus, it is possible that chemotaxis plays a more prevalent role in the overall distribution of *V*. *cholerae* across the length of the intestine than in the penetration from the lumen to the intestinal epithelium. Recent transposon-sequencing (Tn-seq) studies using the infant rabbit show contrasting results with regards to the role of chemotaxis of *V*. *cholerae* in this animal model [42,43]. Fu et al. found that mutants for genes that have chemotaxis-related functions, such as *vspR*, *pomA*, or *cheA*, cause hypercolonization of the infant rabbits [42]. On the other hand, Kamp et al. found that the overwhelming majority of chemotaxis genes are dispensable for infection but played a significant role in the survival of *V*. *cholerae* in pond water [43]. Further work is needed in order to determine the precise role of chemotaxis during *V*. *cholerae* infection.

Bile is a bactericide that appears to act as a chemorepellant driving *V*. *cholerae* out of the intestinal lumen and towards the mucus layer covering the epithelium (Fig 1B and 1C) [44]. *V*. *cholerae* has evolved a very strong avoidance response to bile, as bile significantly increases *V*. *cholerae* motility even at concentrations too low to cause any bactericidal effect (Fig 1B and 1C) [45]. ToxT is a master virulence regulator of *V*. *cholerae* that controls the expression of TCP and the cholera toxin (CT), the main source of the watery diarrhea that causes dehydration [46–50]. Fatty acids found in bile inhibit ToxT activity by binding to its regulatory domain, which prevents ToxT from associating with DNA [45,51–55]. ToxT inhibition by bile suggests a mechanism by which the expression of the virulence cascade would be prevented until the bacterium reaches the appropriate environment. Oppositely, bicarbonate has a positive effect on the virulence cascade of *V*. *cholerae* by increasing the affinity of ToxT for DNA [56–58]. Furthermore, the concentration of bicarbonate lumen versus mucosa is contrary to bile (Fig 1) [56,59,60]. The sum of these factors might allow the proper spatiotemporal pattern of virulence gene expression in the human host.

### Movement through the mucosa

In order to reach the epithelium and deliver CT, *V*. *cholerae* must penetrate a highly viscous mucus layer approximately 150 μm thick, or roughly 50–75 times the body length of *V*. *cholerae* (Fig 1C) [61]. Recent developments support the idea that host mucins act as a physical barrier that *V*. *cholerae* needs to overcome in order to reach the intestinal epithelium [38]. *N*-acetyl-L-cysteine, a mucolytic agent, facilitates *V*. *cholerae* colonization in vivo [38]. In order to break down mucins, *V*. *cholerae* might rely on a mucinase complex, degrading polysaccharide and protein components of mucin in a manner analogous to known processes during *V*. *cholerae* departure from the intestine after infection [62–65]. For example, *V*. *cholerae* produces a soluble mucinase, called haemagglutinin/protease (Hap), which is encoded by *hapA* [62]. In a column assay, expression of *hapA* positively correlates with the capacity of *V*. *cholerae* to move through the mucus layer [63]. As *hapA* is expressed late in infection, it has been suggested that it facilitates detachment from the host epithelium and removal from the mucosa post-infection [66]. However, because mucin induces *hapA* promoter activity [63], it is possible that Hap also facilitates initial penetration of the mucus layer. In addition, some as-yet-undiscovered mucinases might be involved in the early stages of colonization of *V*. *cholerae*.

While a general protease seems to be involved in initial migration through the mucus, *V*. *cholerae* may express specific mucinases near the location where the bacterium preferentially colonizes the intestinal epithelium. Whereas Hap is a metalloprotease that cleaves a wide variety of substrates, TagA, another metalloprotease, may specifically modify mucin glycoproteins attached to the host cell surface [65]. TagA, which is encoded within the Vibrio pathogenicity island (VPI), is expressed and secreted by *V*. *cholerae* under virulence-inducing conditions [65]. As the protein is positively coregulated with TCP and other virulence genes, TagA may play an important role in colonization during the later stages of movement through the intestinal mucosa. Another *V*. *cholerae* virulence factor, neuraminidase (NanH) [67], is an extracellular enzyme that cleaves two sialic acid groups from the GM_1_ ganglioside, a sialic-acid containing oligosaccharide on the surface of epithelial cells, thereby unmasking receptors for CT [68]. As a mucinase with a specific role in infection, NanH may be important in aiding movement through the mucus to the specific site of infection.

## Reversible and Irreversible Attachment

### Finding the preferred site for infection

Once *V*. *cholerae* has penetrated the mucus layer and reached the epithelium, attachment to the epithelial cells likely occurs, since *V*. *cholerae* strains with deletions in genes encoding adhesins show colonization defects in the infant mouse model and in vivo studies demonstrate that *V*. *cholerae* physically interacts with the intestinal epithelium from the early stages of colonization (Fig 1D) [38,69–71]. *V*. *cholerae* produces various nonspecific adhesins that, upon initial contact with the host epithelium, seem to allow the bacterium to determine whether it has reached the appropriate niche without committing to attachment. To our knowledge, adhesins that have been identified in vivo and/or in vitro in *V*. *cholerae* include the flagellum (in addition to its function in motility) [72], Mam7 [73], GbpA [70], OmpU [74], and FrhA (Fig 1D) [71].

Outer membrane adhesion factor multivalent adhesion molecule 7 (Mam7) is one possible example of a nonspecific adhesin involved in *V*. *cholerae* colonization. Loss of Mam7 decreases attachment of *V*. *cholerae* by about 50% in cultured fibroblast cells [73]. Various results suggest the adhesin is nonspecific [73]; Mam7 does not bind to a specific receptor or molecule but instead can establish protein—protein as well as protein—lipid interactions, and Mam7 has been shown to mediate binding to diverse host cells by many gram-negative bacteria. Across pathogenic species, Mam7 is a general adhesion factor that facilitates attachment to various substrates; it is possible that each species also encodes specific adhesins that play a greater role in promoting attachment to unique host cells [73]. Overall, in *V*. *cholerae*, Mam7 likely plays a role in initial attachment to the epithelium (Fig 1D).

Another example of a nonspecific adhesin for *V*. *cholerae* is GlcNAc-binding protein (GbpA), which facilitates attachment to the intestinal epithelium and the chitinaceous surfaces of copepods [70]. GbpA binds specifically to GlcNAc molecules that are attached to glycoproteins and lipids on intestinal epithelial cells and mucus [75,76]. Furthermore, GbpA increases the production of intestinal secretory mucins (MUC2, MUC3, and MUC5AC) in HT-29 intestinal epithelial cells through up-regulation of corresponding genes [75]. However, similar to Mam7, loss of GbpA only decreases attachment in an epithelial cell assay by 50% as compared to wild type [70].

Bacterial outer membrane proteins, which are involved in a wide variety of functions, some of which include attachment, require further investigation as potential nonspecific adhesins in *V*. *cholerae*. In the genus *Vibrio*, outer membrane porins aid in attachment to both biotic and abiotic surfaces [74,77,78]. OmpU plays a role in the attachment of *Vibrio fischeri*, symbiont of the Hawaiian squid *Euprymna scolopes*, to the epithelium of the light organ, and plays a cell line-specific role in the attachment of *V*. *cholerae* to epithelial cells [74,78]. Nonetheless, the possibility that OmpU might play a role in the attachment of *V*. *cholerae* O1 in vivo remains to be determined.

It was recently found, through the use of atomic force microscopy, that *V*. *cholerae* O1 interacts physically with the GM1 ganglioside [79]. The cells show a 5-fold increase in attachment to lipid bilayers coated with GM1 gangliosides compared to control bilayers [79]. Thus, this raises the possibility of NanH and the GM1 ganglioside having several roles in *V*. *cholerae* O1 pathogenesis: (A) NanH releases a carbon source, *N*-acetylneuraminic acid, that confers a competitive advantage to the bacterium in the intestine while unmasking the GM1 ganglioside [80], and (B) the GM1 ganglioside acts as the receptor of CT [67,68] and (C) might act as receptor of a nonspecific adhesin or adhesins.

Attachment to epithelial cells appears to be required in order for *V*. *cholerae* to successfully colonize the SI [70,71,81]. Deletion strains for the adhesins *gbpA* and *frhA* have deficient intestinal colonization in the infant mouse model [70,71]. The effect on colonization of GbpA is particularly striking as, even though it shows just a 50% decrease in attachment in vitro, the mutants show 1-log decrease in colonization of the infant mouse [70]. To date, the effect of Mam-7 in the intestinal colonization of *V*. *cholerae* remains to be elucidated; nonetheless, recent Tn-seq studies did not identify it in their screenings [42,43]. It is possible that nonspecific adhesins such as Mam-7 or GbpA, given their low individual affinity, could act synergistically and that the intestinal colonization defect shown by strains with multiple deletions would be augmented.

The use of transient nonspecific adhesins as early attachment factors in colonization could confer *V*. *cholerae* the advantage of being able to detach from a substrate if it is not conducive to prolonged attachment (e.g., because of the lack of specific nutrients). It is possible that once *V*. *cholerae* attaches to a preferred substrate with nonspecific adhesins, the bacterium could subsequently produce specific adhesins that would allow for committed attachment in a manner analogous to the early stages of biofilm formation on nutrient-rich substrates in the aquatic environment (Fig 1E).

### Committed attachment in chemically favorable conditions

Entering a committed attachment stage remains a possibility in the intestinal colonization of *V*. *cholerae*. Nonetheless, if the bacterium transitions from noncommitted to committed attachment, *V*. *cholerae* must be able to sense specific host signals, such as preferred carbon sources, that would indicate that *V*. *cholerae* has reached the appropriate niche. Recent studies provide evidence for preferential use of specific carbon sources by *V*. *cholerae*. For instance, the ability to utilize two amino sugars abundant in the gut, sialic acid (*N*-acetylneuraminic acid) and GlcNAc (*N*-acetylglucosamine), confers *V*. *cholerae* with a competitive advantage in the infant mouse model of infection [80,82]. Furthermore, ToxT controls the expression of a small RNA, TarA, which influences glucose uptake through its effect on the transcript encoding the glucose transporter PtsG [83]. When the virulence cascade is being expressed, TarA decreases the uptake of glucose because of its negative effect on *ptsG* mRNA [83]. Together, these findings suggest that *V*. *cholerae* has evolved mechanisms to utilize certain carbon sources in the gut mucosa (sialic acid and GlcNAc) in a preferential manner over others (glucose). Although evidence indicates favored use of certain carbon sources by *V*. *cholerae* and thus supports the notion that the bacterium would delay committed attachment until reaching chemically favorable conditions for virulence, no adhesins involved in committed attachment are known in *V*. *cholerae*, and the existence of this stage during intestinal colonization remains hypothetical. Once the virulence cascade is activated, the attachment of *V*. *cholerae* to intestinal epithelial cells increases [69]. A possible way to identify specific adhesins involved in committed attachment might be to ectopically express *toxT* in different mutant strains and identify those that attach similarly to the control strains and thus do not experience an increase in their attachment to epithelial cells.

## Final Stages of Colonization

### Proliferation and microcolony formation

After attachment to the intestinal epithelium, the bacterium decreases motility [84], begins to proliferate, and initiates the virulence cascade (Fig 1F). *V*. *cholerae* forms TCP-mediated clusters of bacterial cells called microcolonies (Fig 1G). It was recently shown that microcolonies originate from single cells after reaching the intestinal epithelium (Fig 1G) [38]. To date, several roles of the pilus have been determined: TCP enhances attachment to intestinal epithelial cells and facilitates bacteria—bacteria interactions, visualized in vitro as autoagglutination, by tethering the cells together; the ability to form microcolonies correlates with the ability to colonize the infant mouse and humans [23,85]. TCP acts as the receptor of the CTX phage, a filamentous bacteriophage that encodes CT [86]. Interestingly, an in-frame deletion mutant for *tcpA* shows highly reduced expression of the gene encoding the major subunit of CT in vivo, indicating that the presence of an intact TCP apparatus appears to be essential for effective regulation of the virulence cascade [81]. TCP is also required for the secretion of the soluble colonization factor TcpF [87]. In vivo, a *tcpF* mutant is severely defective for colonization, a reduction equivalent to the effect seen with a *tcpA* mutant, which encodes the major pilin subunit [87]. Although TcpF mutants are still able to form microcolonies, they are loosely packed and have decreased adherence around the edges; thus, it appears that TcpF functions as an enhancer of microcolony formation in vitro [69].

Forming microcolonies within the host may also be beneficial to *V*. *cholerae* for other reasons, including more efficient nutrient uptake and protection from antimicrobials like bile or bactericidal compounds produced near the intestinal epithelium [69,85]. Furthermore, it is thought that microcolonies might protect *V*. *cholerae* from being shed [38]. In strains with functional quorum-sensing systems, virulence is repressed at high cell density [66]. However, quorum sensing does not seem to play an essential role in virulence, as various toxigenic strains of *V*. *cholerae* have a naturally occurring frameshift mutation in the *hapR* gene, which encodes the master regulator of quorum sensing [66].

## Synthesis and Next Steps

The detailed mechanisms facilitating intestinal colonization of bacterial pathogens are beginning to be understood. In this perspective, we provide a comprehensive model that draws upon recent findings in the field and proposes a series of steps that appear to be necessary for *V*. *cholerae* to effectively colonize the intestinal epithelium (Fig 1). Models such as the one described here might provide researchers with ways to generate testable hypotheses, furthering the knowledge of the field. Some areas of the intestinal colonization dynamics of *V*. *cholerae* covered in this model that need further exploration include the roles of the chemical gradients of bile and bicarbonate on *V*. *cholerae* virulence gene expression, the variable distribution of components of the mucus throughout the SI and the enzymes involved in its degradation, the specific role, if any, of chemotaxis during infection, the conditions necessary for prolonged attachment, and the confirmation and identification of specific adhesins.
